# Mortality of older persons with and without abnormalities in the physical examination of arterial system

**DOI:** 10.1007/s40520-022-02232-7

**Published:** 2022-08-22

**Authors:** Jarosław Królczyk, Karolina Piotrowicz, Anna Skalska, Małgorzata Mossakowska, Tomasz Grodzicki, Jerzy Gąsowski

**Affiliations:** 1 Department of Internal Medicine and Gerontology, Jagiellonian University Medical College, 2 Jakubowskiego St., 31-501 Krakow, Poland; 2 University Hospital in Krakow, Krakow, Poland; 3 Study On Ageing and Longevity, International Institute of Molecular and Cell Biology, Warsaw, Poland

**Keywords:** Peripheral arterial disease, ABI, Physical examination, Screening

## Abstract

**Background:**

Ankle-brachial index (ABI) is a screening tool for peripheral arterial disease (PAD). However, persons with normal ABI may still exhibit abnormalities in the physical examination of arterial system (PHEA).

**Objective:**

In older persons from the PolSenior study, we aimed to assess the risk of total mortality associated with abnormalities in PHEA in the context of dichotomised ABI.

**Methods:**

We used data from the PolSenior survey and matched them with mortality information from the Polish Census Bureau. We obtained sociodemographic, medical history, and lifestyle data. The PHEA by a geriatrician included carotid, femoral, popliteal, posterior tibial and the dorsalis pedis arterial pulses, and auscultation of aorta, carotid, femoral, and renal arteries. Ankle-brachial index was tibial to brachial SBP ratio. We plotted the stratified Kaplan–Meier curves and used Cox’s regression to assess the unadjusted and adjusted influence of PHEA result on time to death.

**Results:**

The mean (standard deviation, SD) age of 852 persons (46.7% women) was 74.7 (10.6) years. In the ABI < 0.9 group, the PHEA was not associated with mortality. However, in the ABI ≥ 0.9 group, both in unadjusted and adjusted (RHR; 95% CI: 1.08; 1.02–1.16, *p* = 0.01) Cox regression, PHEA greater by 1 score was associated with mortality. Presence of 4 or more PHEA abnormalities was raising the risk in the ABI ≥ 0.9 group to the level associated with ABI < 0.9.

**Conclusions:**

In the older persons with normal ABI, the greater number of abnormalities during physical examination of arteries may be indicative of higher risk of death.

## Introduction

Physical examination remains an important part of the patient’s assessment, despite the rapid changes in medical technology. The physical examination of the arterial system (PHEA) requires training and experience, is time consuming, and in some instances may be subjective. Nevertheless, the PHEA findings may provide valuable information on the presence of peripheral arterial disease (PAD) and underlying atherosclerosis even in apparently healthy subjects [1–5].

With the advent of novel techniques, there is a tendency to supplant PHEA with such modalities as ultrasonography, computed tomography, and magnetic resonance imaging [6]. This is especially true in the rapidly changing world where the growing lack of the experienced health care professionals will be a major concern. On the other hand, the physical examination does not require special setting and equipment and can be performed by a single examiner. As an extension, techniques such as the estimation of the ankle-brachial index (ABI) based on the systolic blood pressure measured on brachial and tibial arteries, may enhance the predictive value of physical examination of arteries [7, 8]. ABI < 0.9 is a sign of PAD, 1.4 > ABI ≥ 0.9 is considered normal, while ABI ≥ 1.4 corresponds to stiff arteries. In earlier research, we did not demonstrate 9-year difference in mortality between the 1.4 > ABI ≥ 0.9 and the ABI ≥ 1.4 groups of older persons [9]. Previously, we demonstrated the age dependent fashion for the relation between ABI and mortality in older persons [9]. We also found that the abnormalities during PHEA are prevalent in persons with greater ABI [10]. However, the relation between PHEA and mortality in the context of ABI has not been assessed.

We hypothesize, that on top of the ABI classification, PHEA may add to the prediction of mortality in older adults. Accordingly, we have now assessed the 9-year risk of mortality associated with the result of PHEA in the context of dichotomised ABI, in a cohort of older persons from the PolSenior study.

## Methods

### Study population

We included data of 852 participants from the PolSenior study. PolSenior was a nationwide, multicentre, cross-sectional survey of health status and its determinants in the community dwelling subjects aged 55–59 and 65 and more years carried out between January 2009 and April 2010. The methodology for selecting study participants has been described in detail previously [11]. Briefly, the individuals were randomly selected from 16 voivodships of Poland using three-stage, proportional sampling process stratified by gender and age group. The response rate was 42% and ranged from 32 to 61% between provinces. The 5695 participants were examined using a structured questionnaire conducted by pretrained nurses at participants’ home. According to the protocol, approximately 20% of this group (*n* = 1018) were examined by a geriatrician. The examination by geriatrician was offered in nine of sixteen provinces in Poland in which the availability of geriatricians made the examination possible. The unselected participants, both from urban and rural areas, were contacted and those who agreed to participate were examined. In 852 participants, the complete vascular data were available. We used the survival data until 20th of April 2019. The study was approved by the Bioethics Committee of the Medical University of Silesia in Katowice (KNW-6501-38/I/08). Informed consent to take part was obtained from the participants.

### Data collection

We gathered information concerning the sociodemographic data, past medical history, information about smoking habits using a structured questionnaire administered by trained nurses during home visits. The PHEA by the geriatrician included palpation of carotid, radial, femoral, popliteal, posterior tibial and the dorsalis pedis arterial pulses, and auscultation of aorta, carotid, femoral, and renal arteries. The absence of pulse or presence of murmur on a given site were each assigned a score of one. The sum of the results (minimal value 0, maximal value 17) from the sites given above constituted the PHEA result.

Ankle-brachial index was measured in accordance with guidelines [6]. After 5-min rest, the supine blood pressure was measured three times consecutively on right brachial artery with an A&D, UA 787 Plus automatic device. An average of second and third readings were used in the analyses. The systolic blood pressure (SBP) on both posterior tibial arteries was assessed using Doppler Bidop ES-100VX device with 8 MHz ultrasound probe (Hadeco Inc., Kawasaki, Japan). The cuff was positioned on a calf with the distal edge of the cuff 3 cm above the medial malleolus. The SBP measurement was performed on both lower extremities. ABI was calculated as systolic blood pressure on posterior tibial artery (mm Hg)/systolic blood pressure on brachial artery (mm Hg). The average difference between right and left lower extremity values was not statistically significant (*p* = 0.27). There were five missing values for the left lower extremity. Accordingly, we decided to use the values from right lower extremity in all subsequent analyses. For the stratified analyses, ABI was categorized into low: < 0.9, and normal/high: ≥ 0.9.

### Acquisition of covariates

Body mass index (BMI) was calculated according to the formula: body weight (kg)/height^2in^ (m^2^). Creatinine and cholesterol levels were measured by the standard method with an automated analyzer Modular PPE (Roche Diagnostics) and Roche Diagnostics GmbH reagents (Mannheim Germany). A serum aminoterminal pro-brain natriuretic peptide (NT-proBNP) level was measured by an electrochemiluminescence immunoassay (ECLIA) method (Roche Diagnostics GmbH, Mannheim, Germany) with a immunoassay analyzer Cobas e411 (Roche Diagnostics GmbH, Mannheim, Germany). Plasma interleukin 6 (IL-6) concentration was determined by high-sensitivity ELISA using R&D Systems kits (Minneapolis, MN, USA). High-sensitivity CRP (hsCRP) levels were measured using a high-sensitivity immunoturbidimetric method (Modular PPE, Roche Diagnostics GmBH, Mannheim, Germany, sensitivity 0.11 mg/L).

Data on deaths over 9 years after beginning of the study were obtained from Universal Electronic System for Registration of the Population.

### Statistical methods

The statistical analyses were performed with SAS 9.4 (SAS Institute Inc., Cary, NC, USA). The means were compared using standard normal *Z*-test, proportions with chi-square test. In the survival analyses, we charted the Kaplan–Meier curves with log-rank test and fitted the unadjusted and adjusted Cox regression models. The Kaplan–Meier curves were plotted by the PHEA score within either 0–3 or ≥ 4. We obtained the PHEA cut-off value of 4 by running several models with increasing PHEA cut-offs from 1 to 6. At the value of 4, we observed a phase change showing the difference in survival, whereas no further change was observed by increasing the cut-off up to the value of 6. The hierarchical adjustment of Cox models included sex, and age, then additionally either the diabetes mellitus, smoking, BMI, SBP, and LDL-cholesterol or NT-proBNP, IL-6, CRP, and creatinine. In the models adjusted for the larger sets of confounders, we first chose the available classic modifiable risk factors for atherosclerosis and then the biochemical biomarkers with the potential of being associated with atherosclerotic disease. Finally, in the groups with ABI ≥ 0.9, we ran a model containing all variables that were significant in the previous analyses. All p values are two-sided with significance at 5%.

## Results

### Group characteristics

The mean (standard deviation, SD) age of 852 participants (46.7% women) was 74.7 (10.6) years. There were 118 (13.9%) persons with the ABI < 0.9 and 734 (86.2%) with ABI ≥ 0.9. The baseline characteristics are contained in Table 1. Participants with ABI < 0.9 were older, and more often were active smokers. The systolic (SBP) and diastolic (DBP) blood pressures averaged 154.8 (22.5) vs. 143.2 (20.5) (*p* < 0.0001) and 80.9 (11.5) mmHg vs. 80.5 (11.5) mm Hg (*p* = 0.73), ABI < 0.9 and ABI ≥ 0.9 groups, respectively. These two groups did not differ in LDL cholesterol levels. The ABI < 0.9 participants had higher concentration of creatinine, N-terminal pro-brain natriuretic peptide (NT-proBNP) and inflammatory markers, hsCRP and IL-6. In this group, more clinical vascular abnormalities such as absence of pulses or presence of vascular murmurs were found. In both groups, the most common chronic disease was arterial hypertension, but it was more frequent in participants with ABI < 0.9. In contrast, the prevalence of diabetes, myocardial infarction, stroke, and heart failure did not differ between the groups. Moreover, the average survival time of people with ABI < 0.9 was significantly shorter.

**Table 1:** Characteristics of the study group depending on the ABI value ≥ 0.9 and < 0.9

Parameter	ABI < 0.9 *n* = 118 (mean (SD)) or percentage [*n*]	ABI ≥ 0.9 *n* = 734 (mean (SD)) or percentage [*n*]	*p*
Age (years)	79.6 (10.0)	73.9 (10.5)	< 0.0001
Women, % [*n*]	36.4 [43]	48.4 [355]	0.02
ABI right side	0.68 (0.2)	1.17 (0.2)	< 0.0001
ABI_left side	0.79 (0.2)	1.14 (0.2)	< 0.0001
SBP (mmHg)	154.8 (22.5)	143.2 (20.5)	< 0.0001
DBP (mmHg)	80.9 (11.5)	80.5 (11.5)	0.73
ADL (points)	5.4 (1.1)	5.8 (0.7)	0.0004
BMI (kg/m^2^)	27.4 (5.2)	28.4 (4.9)	0.03
Smokers, current, % [*n*]	22.0 [26]	9.9 [73]	0.0001
Survival time (years)	6.0 (3.0)	7.7 (2.7)	< 0.0001
NT-proBNP (pg/ml)	766.6 (1624.6)	365.5 (654.7)	0.01
Creatinine (mg/dl)	1.1 (0.4)	0.9 (0.3)	0.0007
LDL cholesterol (mg/dl)	116.4 (39.4)	119.8 (39.1)	0.40
hsCRP (mg/l)	5.7 (8.5)	3.9 (7.8)	0.02
IL-6 (pg/ml)	4.6 (6.1)	2.7 (2.5)	0.002
PHEA	2.4 (2.7)	0.9 (1.7)	< 0.0001
Hypertension, history of, % [*n*]	83.9 [99]	67.8 [498]	0.0005
Diabetes mellitus, history of, % [*n*]	23.7 [28]	17.2 [126]	0.09
Myocardial infarction, history of, % [*n*]	16.1 [19]	10.5 [77]	0.08
Stroke, history of, % [*n*]	10.2 [12]	6.0 [44]	0.09
Heart failure, history of, % [*n*]	8.5 [10]	7.9 [58]	0.83

*ABI* ankle-brachial index; *SBP* systolic blood pressure; *DBP* diastolic blood pressure; *ADL* activities of daily living scale; *BMI* body mass index; *NT-proBNP* N-terminal pro-brain natriuretic peptide; *LDL* low-density lipoprotein; *hsCRP* high-sensitivity assay C-reactive protein; *IL-6* interleukin 6; *PHEA* PHysical Examination of the Arterial system abnormalities

### The abnormalities in the physical examination of arteries

**Fig. 1:**
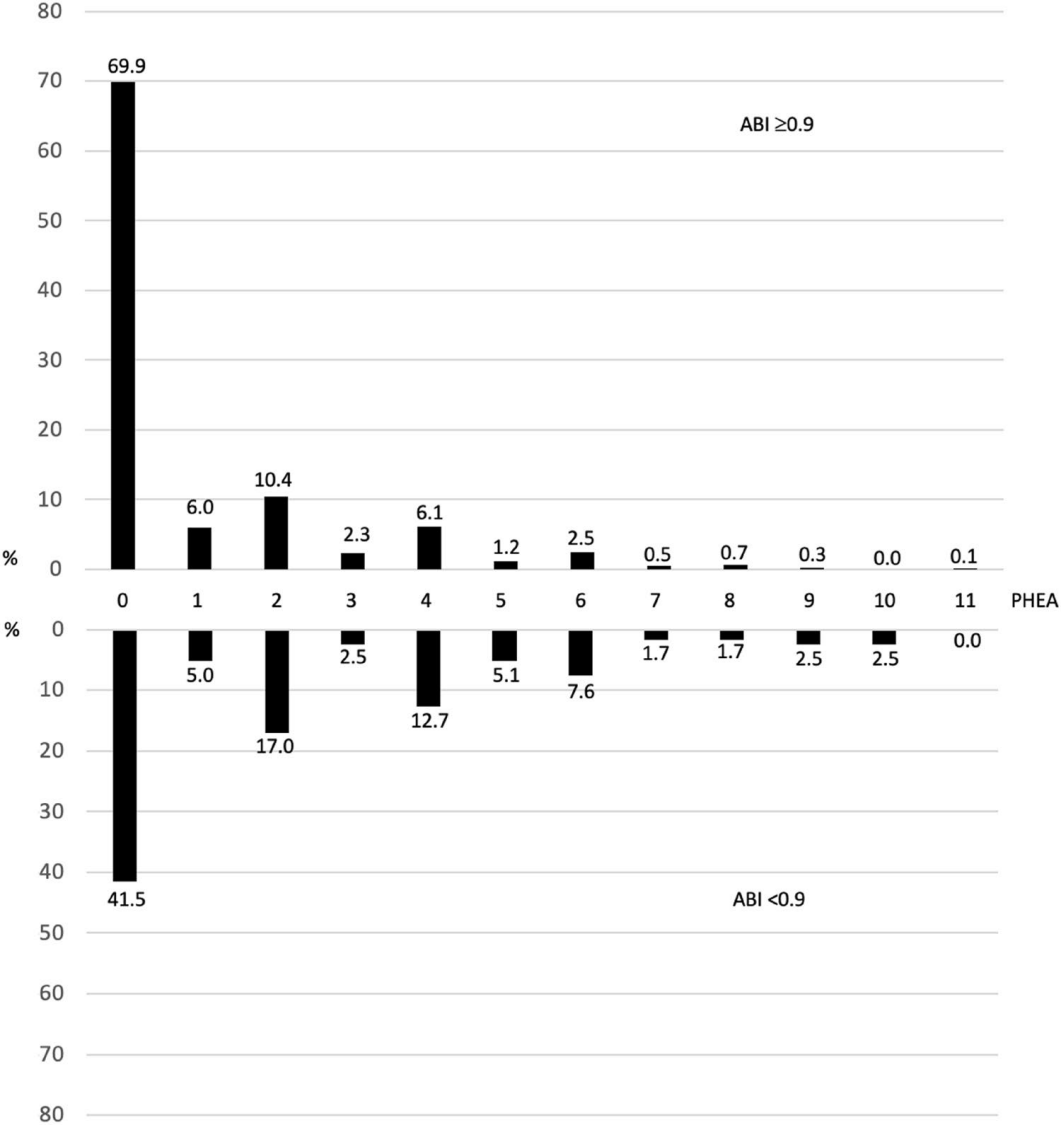
The frequency of abnormalities in physical examination of arteries (PHEA) in the ABI < 0.9 and ABI ≥ 0.9 groups

Overall, the persons within ABI < 0.9 had more vascular abnormalities and thus higher PHEA score. Only 41.5% of them had PHEA = 0. Of the ABI ≥ 0.9 group, 69.9% had PHEA 0. PHEA ≥ 4 was present in 33.9, and 11.4% of the ABI < 0.9 and the ABI ≥ 0.9 groups, respectively (*p* < 0.0001). The details concerning the abnormalities in physical examination are presented in Fig. 1.

### Survival analysis

To establish factors independently associated with overall mortality in analyzed older population, we built Cox regression models separately for the group with ABI < 0.9 and ABI ≥ 0.9. Results of regression analysis are presented in Table 2.

**Table 2:** Physical examination of the arterial system abnormalities and mortality by ABI categories

Model	ABI < 0.9 *n* = 118 RHR (95% CI)	*p*	ABI ≥ 0.9 *n* = 734 RHR (95% CI)	*p*
Unadjusted				
PHEA	1.08 (1.00–1.16)	0.05	1.13 (1.08–1.20)	< 0.0001
Adjusted for age, sex				
PHEA	1.00 (0.92–1.10)	0.89	1.07 (1.01–1.14)	0.02
Age	1.08 (1.05–1.11)	< 0.0001	1.12 (1.10–1.13)	< 0.0001
Male sex	1.19 (0.76–1.90)	0.45	1.50 (1.19–1.90)	0.0006
Adjusted for age, sex, diabetes, smoking, BMI, SBP, LDL cholesterol				
PHEA	1.02 (0.92–1.13)	0.67	1.08 (1.02–1.16)	0.01
Age	1.10 (1.06–1.14)	< 0.0001	1.12 (1.10–1.14)	< 0.0001
Male sex	1.27 (0.74–2.16)	0.38	1.38 (1.07–1.78)	0.01
Diabetes	0.72 (0.37–1.40)	0.34	0.97 (0.68–1.38)	0.85
Smoking	1.12 (0.61–2.05)	0.72	1.94 (1.26–3.00)	0.003
BMI	1.05 (0.99–1.11)	0.12	0.96 (0.94–0.99)	0.01
SBP	0.99 (0.98–1.00)	0.08	1.01 (1.00–1.01)	0.05
LDL cholesterol	1.00 (1.00–1.01)	0.32	1.00 (1.00–1.00)	1.00
Adjusted for age, sex, NT-pro-BNP, Il-6, hsCRP, creatinine				
PHEA	1.02 (0.93–1.11)	0.72	1.09 (1.02–1.16)	0.01
Age	1.09 (1.05–1.12)	< 0.0001	1.10 (1.09–1.12)	< 0.0001
Male sex	0.99 (0.57–1.71)	0.97	1.49 (1.14–1.93)	0.003
NT-proBNP	0.98 (0.82–1.18)	0.86	1.41 (1.23–1.63)	< 0.0001
IL-6	0.98 (0.93–1.04)	0.51	1.08 (1.03–1.13)	0.001
hsCRP	1.04 (1.00–1.08)	0.03	1.00 (0.99–1.12)	0.88
Creatinine	1.37 (0.67–2.79)	0.39	1.14 (0.72–1.81)	0.58

Results of the unadjusted and adjusted Cox regression analysis models

RHRs are per 1 unit change. For units, see Table 1

*ABI* ankle-brachial index; *PHEA* PHysical Examination of the Arterial system abnormalities; *BMI* body mass index; *SBP* systolic blood pressure; *LDL* low-density lipoprotein; *NT-proBNP* N-terminal pro-brain natriuretic peptide; *IL-6* interleukin 6; *hsCRP* high-sensitivity assay C-reactive protein

In all analyses that we performed, unadjusted, adjusted for age and sex, and adjusted for other confounders, the PHEA abnormalities were not associated with 9-year total mortality in the ABI < 0.9 group. In the ABI ≥ 0.9 group, PHEA abnormality greater by 1 score was associated with greater risk of death (RHR 1.13 [1.08–1.20], *p* < 0.0001, to 1.08 [1.02–1.16], *p* = 0.01, unadjusted and fully adjusted model, respectively). After the adjustment for all confounders that in the previous models were almost significantly associated with the risk of mortality, in the group with ABI ≥ 0.9 the PHEA greater by 1 score was associated with 7% greater risk of death (RHR 1.07 [1.00–1.14], *p* = 0.05).

**Fig. 2:**
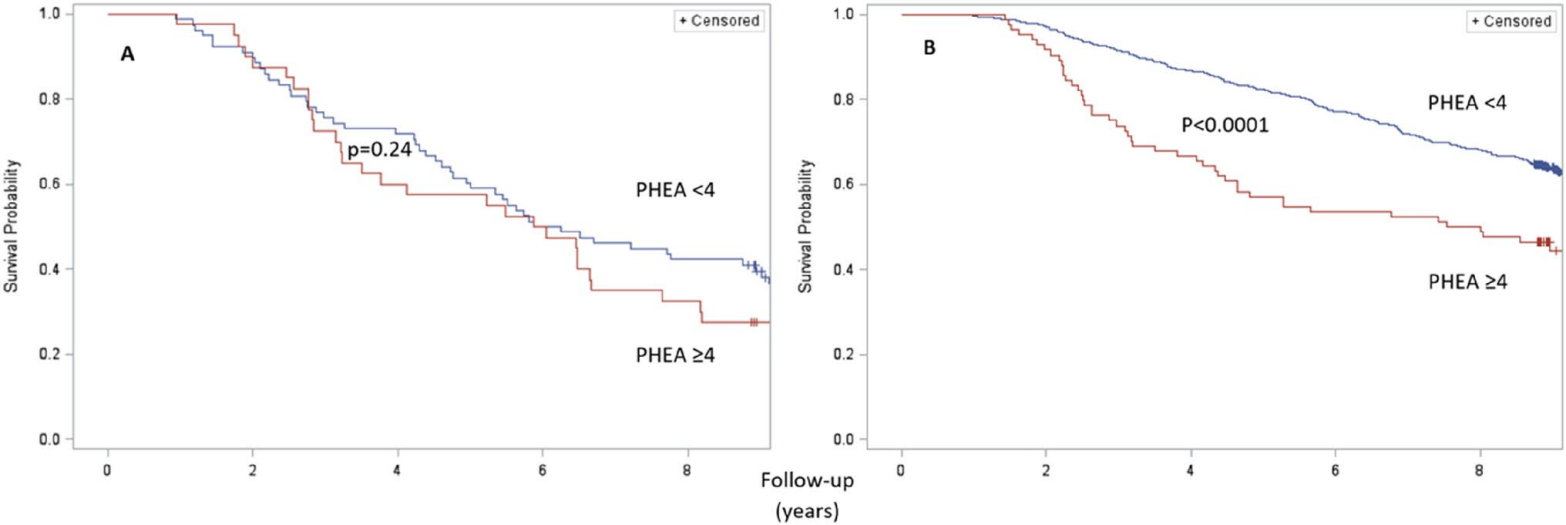
Nine-year probability of survival in groups with PHEA < 4 or ≥ 4. Panel **A** ABI < 0.9, panel **B** ABI ≥ 0.9. The *p* values were derived from log-rank test. *ABI* ankle-brachial index; *PHEA* physical examination of arteries

The fitting of the Kaplan–Meier curves demonstrated that the 9-year mortality of individuals with ABI ≥ 0.9 was influenced by the PHEA score of 0–3 or ≥ 4 (*p* < 0.0001), but the mortality in persons with ABI < 0.9 was not influenced by such defined PHEA score status (*p* = 0.24) (Fig. 2).

## Discussion

We found that in older community dwellers, ABI status modifies the prognostic significance of the physical examination of arterial system. In older persons with ABI ≥ 0.9, the presence of 4 or more abnormalities on arterial physical examination (PHEA) offsets the benefit of better (≥ 0.9) ABI. Further, in persons with ABI ≥ 0.9, in the Cox’s models, before and after adjustment for confounders, the relative risk of mortality increased with accretion of abnormalities in physical examination of arterial system. No such relation was present in the group with ABI < 0.9.

Peripheral artery disease affects approximately 230 million people worldwide and is associated with increased rates of total and cardiovascular morbidity and mortality [12, 13]. Physical examination is the basis of the clinical evaluation of the patient. The essential part of physical examination is palpation and auscultation of the arteries. The lack of pulse or presence of murmurs suggests the existence of peripheral atherosclerosis, which may be associated with a need for vascular intervention, and further the risk of limb amputation and death [14]. On the other hand, the absence of abnormalities, especially presence of normal lower extremity pulses, has been associated with high negative predictive power, reaching 98% [15]. The presence of peripheral arterial atherosclerosis usually means that atherosclerosis is widespread, potentially affecting also the coronary and cerebral circulations [16, 17].

ABI has been advocated as a next step between the clinical diagnosis and angiographic studies [12]. However, none of the guidelines specify the extent to which abnormalities in the physical examination translate into the actual justification for extending costly vascular diagnostics. For example, in the US Medicare, in 2001, a total of $4.37 billion were spent on PAD-related treatment. Eighty-eight percent of the expenditure covered the in-hospital care, and treatment costs increased with age [18]. According to the result from the LIBERTY 360° Trial, the cost increases also with increasing Rutherford classification [19].

In our study, we have demonstrated that in people with ABI < 0.9, that is with established atherosclerosis, the number of vascular abnormalities on physical examination had no prognostic significance. The only factors associated with a higher risk of death in this group were age and CRP. This underlines the extent of risk associated with developed PAD [14]. On the other hand, in the group with ABI ≥ 0.9, not only the classic risk factors such as age, male sex, smoking, higher SBP, but also the levels of IL-6 and NT-proBNP increased the risk of death which is in line with the results of earlier studies [20–24]. This may indicate a potential role of inflammageing and subclinical circulatory failure as clinical modifiers of PAD [24, 25].

It was previously shown that even in persons with ABI < 0.9, 70 to 90% of PAD cases are asymptomatic or the symptoms do not follow the classic pattern of claudication [12].

We have shown that in the group with ABI ≥ 0.9, the greater PHEA had prognostic significance. The risk increased markedly when 4 or more abnormalities on arterial physical examination had been present (Fig. 2). This can help identify additional patients at risk, as most cases of peripheral atherosclerosis in asymptomatic [6, 12, 26].

Performance of the ABI is relatively simple, quick, reproduceable, inexpensive, and with some training may be performed by a technician-level staff. Both our data and the data cited above imply that its routine performance helps identify those at risk of mortality and the decrease of the disability-adjusted life years. The performance of full vascular physical examination is more time-consuming, is performed by the physician, and requires both under-graduate training and post-graduate experience. Our data indicate, that in older persons with ABI ≥ 0.9, the performance of physical examination of arterial system may further identify persons who despite ABI ≥ 0.9 might benefit from more aggressive approach.

Persons with ABI < 0.9 are usually promptly redirected to further imaging studies. However, of the persons with ABI ≥ 0.9, those with 4 or more vascular abnormalities on physical examination may have the level of risk of mortality similar to the risk associated with ABI < 0.9. Based on literature, we hypothesize that individuals with ABI ≥ 0.9 and 0–3 PHEA abnormalities, would be candidates for lifestyle modification, treatment as per other indications and the re-evaluation after 1 year [26, 27].

Our results need to be considered in the context of the study limitations. First, our results are based on a cross-sectional data matched with the mortality data obtained after 9 years. On the other hand, our cohort was drawn in a pre-planned manner as a part of a study representative at a national level. Second, as the outcome, we use total mortality, and we lack the possibility to extract the reasons for death or the non-fatal events. However, total mortality is an important outcome in a population at the mean age at which an average lifespan is estimated as 10 years [28]. In our study, we pooled together the data of persons with 0.9 ≤ ABI ≤ 1.4 and ABI > 1.4. We did that because in our earlier analyses, the Kaplan–Meier curves for survival in these groups did not differ [9]. In our group, persons with ABI < 0.9 have higher SBP values than persons with ABI ≥ 0.9. This might raise the question of the blood pressure control. Further still, we did not have the access to the medication status. However, with the ABI < 0.9 group representing 13.8% of the entire group and given that the prevalence of hypertension is 83.4% in the ABI < 0.9 and 68% in the ABI ≥ 0.9 groups, our rates of blood pressure control do not seem to deviate from what is generally observed.

In conclusion, ABI may help to identify older persons at greater risk of death mediated by the presence of potentially widespread atherosclerosis. Individuals with ABI ≥ 0.9 may especially benefit from the performance of full vascular physical examination. Such examination may, on top of ABI, help further identify persons at greater risk of mortality. Based on our results, we propose a sequential screening approach where ABI could be performed by the trained nurse first, followed by the detailed physical examination especially in persons with ABI ≥ 0.9. However, our results need independent confirmation and assessment.
